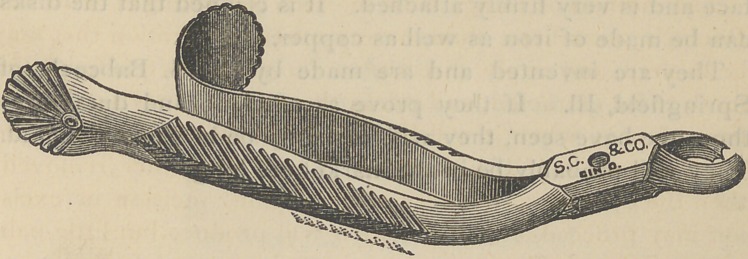# Editorial

**Published:** 1876-12

**Authors:** 


### Editorial.

### LOCAL ANÆSTHESIA.

Many methods of attaining local anæsthesia have been devised and employed, That of reducing the temperature of the part to be operated upon to that point at which sensitiveness no longer exists. This was, and sometimes is yet, done by the application of ice, ether spray, etc., but other methods of more convenient application are now used. Various preparations have been suggested for this purpose. The following we have used for a number of years, viz: a mixture of chloroform, tinctures of aconite, belladonna and opium. This applied to the gums for a few moments, will, in many cases, very much obtund sensibility, and in some relieve it altogether, so that a tooth may be extracted, or a deep incision made without pain.

The dental pain obtunder which was introduced to the profession about three years ago, for relieving sensitive dentine, is good as a local anæsthetic. Another preparation, prepared and introduced by Dr. Von Bonhorst, of Lancaster, Ohio. This is also quite efficient. Dr. Von Bonhorst has invented an instrument for using his preparation, or any other, for like purpose. It consists of two small metallic cups, for holding sponges, attached to the ends of a broad wire, which is about seven inches long; this is the handle of the appliance. Its form is accurately shown by the following cut.

The method of using, is to moisten the sponges with the Doctor's anæsthetic fluid, or any other preparation that may be desired, and apply directly on the gum, each side of the tooth to be extracted, and retain there from thirty seconds to two minutes, when it will be found that the sensibility of the tooth is very much diminished, if not altogether removed; then the operation, whether of extraction, incision or excision may proceed, either of which will produce but little pain to the patient. This is a very convenient form of instrument and it should be in the possesion of every dentist.

A little single holder for the spongue accompanies the instrument; this is for application to an abscess, when cutting is required at only one point.

We have found the application very valuable in many instances.

### A NEW FORCEPS.

The accompanying cut represents a forceps for the extraction of the left inferior molars, invented by Prof. Watling of Michigan University Dental College. It is designed to take the place of the ordinary forceps for these teeth. It has the general form of the right inferior molar forceps; its handle is a little longer in its curve and reach than that instrument; its grasp upon and relation to the tooth for which it is designed, is about the same as that of the forceps for the right side.

The force for the operation is far better applied and directed, and greater too if need be, than with the common forceps.

It is so efficient that the Doctor has denominated it the "University Forceps."

### DIAMOND DISKS.

We have recently received disks made of copper and coated with diamond dust, similar in size and form to the corrundum disks, they are to be used for the same purpose, viz., separating and dressing the natural teeth. They are in some respects better than the corundum disks; in the first place they can be made thinner; in the next place they will not break, and again, they have sufficient spring or elasticty to approach and accommodate themselves to points and localities that the corundum wheels can not reach; and again, they will wear much longer than those in common use. How long they will wear has not been ascertained; they have been in use about six months without any apparent deterioration; the supposition is that they will last for years. And in the last place they leave a much smoother surface than the corundum. They may be used for all purposes the corundum disks are used.

Strips of copper set or lined with the diamond dust are made for polishing the proximate surfaces of the teeth and fillings. They are made of various widths and thicknesses. Of the method of attaching the diamond dust to the copper we are not advised, but it seems to be wrought into the surface and is very firmly attached. It is claimed that the disks can be made of iron as well as copper.

They are invented and are made by Dr. S. Babcock, of Springfield, Ill. If they prove as efficient and durable as those we have seen, they will certainly be in great demand. They will probably be in the market ere long.

## Figures and Tables

**Figure f1:**
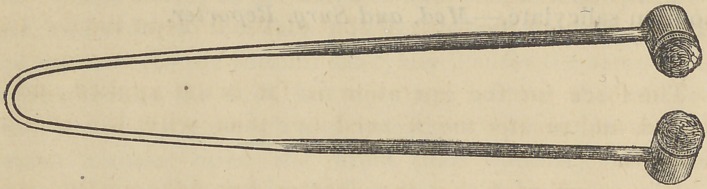


**Figure f2:**